# An evaluation of antibiotic options for the treatment of biothreat pathogens

**DOI:** 10.3389/frabi.2025.1611588

**Published:** 2025-08-14

**Authors:** J. Matthew Meinig, Michelle Nelson, Christopher K. Cote, Stevan R. Emmett, Sarah V. Harding

**Affiliations:** ^1^ Bacteriology Division, United States Army Medical Research Institute of Infectious Diseases, Frederick, MD, United States; ^2^ Defence Science and Technology Laboratory, Porton Down, Salisbury, United Kingdom; ^3^ Respiratory Sciences, University of Leicester, Leicester, United Kingdom

**Keywords:** antibiotics, biodefence, medical countermeasures, antimicrobial susceptibility, biocontainment

## Abstract

The development of medical countermeasures against pathogens of biodefense concern remains critical to protecting military and public health. This review compares data detailing antibacterial activity and efficacy for a selection of antibiotics evaluated against potential bacterial biothreat pathogens. The human safety and tolerability of these formulations were also considered. This review includes finafloxacin, levofloxacin, delafloxacin, omadacycline, gepotidacin, tebipenem and sulopenem. The selection criteria of these antibiotics were 1) the availability of an oral formulation, 2) the regulatory status (licensed by a regulatory authority or in an advanced stage of development) and 3) the availability of publicly available information on the biodefence pathogens of concern. We hope to highlight approved or advanced clinical candidates that have significant and unique potential in the biodefense space which may be deployed to protect both the public and warfighter against these bacterial infections.

## Introduction

Effective and efficient biodefence strategies can be addressed, in part, through the use of broad spectrum antibiotics to provide an enhanced treatment capability against potential bacterial biothreat pathogens. These pathogens may include *Yersinia pestis*, *Francisella tularensis*, *Burkholderia pseudomallei*, *Burkholderia mallei*, *Bacillus anthracis*, and *Coxiella burnetii*, which cause the diseases plague, tularaemia, melioidosis, glanders, anthrax and Q fever, respectively^1^ ^,^ ^2^. They can be challenging to treat, particularly when patients have severe symptoms, and advanced disseminated disease, sepsis, or chronic infection, all of which require efficacious and lengthy courses of antibiotics. Current treatments for these infections include ciprofloxacin and levofloxacin (e.g., plague, tularaemia, anthrax), gentamicin (e.g., plague, tularaemia), doxycycline (e.g., plague, tularaemia, anthrax, Q fever) and ceftazidime/meropenem with co-trimoxazole/co-amoxiclav (e.g., melioidosis, glanders) (Van Zandt et al., 2013; Nelson et al., 2021; Bower et al., 2023; Currie et al., 2023; Nelson et al., 2024).

Seven antibiotics were selected for review based on the availability of an oral formulation and their licensure status by the Food and Drug Administration (FDA), the European Medicines Agency (EMA), or the Medicines and Healthcare Products Regulatory Agency (MHRA), either being licensed or close to being licensed for a non-biothreat clinical indication. Antibiotics with oral formulations were selected as they can be self-administered without the need for in-patient care. An open-source literature review was performed, identifying published *in vitro* antibacterial activity and *in vivo* efficacy data for the fluoroquinolones finafloxacin, delafloxacin and levofloxacin, the tetracycline omadacycline, the triazaacenaphthylene gepotidacin and the β-lactams tebipenem and sulopenem (**Table 1**). The literature reviewed included published manuscripts. We recognise other unpublished data may have been generated which is not accessible and is therefore excluded from this review.

**Table 1:** The clinical status of the antibiotics discussed in this review.

Antibiotic and brand name	Antibiotic class	Mechanism of activity	Developer	Available formulations	Licensed	Regulator	Indication
Finafloxacin otic suspension (Xtoro)	Fluoroquinolone	DNA replication inhibitor	MerLion Pharmaceuticals	IV, oral, otic suspension	Otic suspension only	FDA, Health Canada	Acute otitis externa^#^
Delafloxacin (Baxdela)	Fluoroquinolone	DNA replication inhibitor	Melinta Therapeutics	IV, oral	Yes	FDA, EMA	CABP and ABSSSIs^#^
Levofloxacin (Levaquin)	Fluoroquinolone	DNA replication inhibitor	Sanofi-Aventis	IV, oral,	Yes	FDA	Wide ranging including respiratory infections, urinary tract infections, meningitis, anthrax, plague Treatment of *Pseudomonas aeruginosa* infections in CF patients
Omadacycline (Nuzyra)	Tetracycline	Protein synthesis inhibitor	Paratek Pharmaceuticals	IV, oral	Yes	FDA	CABP and ABSSSIs^#^
Gepotidacin (Blujepa)	Triazaacenaphthylene	DNA replication inhibitor	GSK	IV, oral	Oral only	FDA	uUTI^##^
Tebipenem pivoxil hydrobromide (Orapenem)	Beta lactam	Cell wall synthesis inhibitor	Spero Therapeutics	Oral prodrug	No	N/A	N/A
Sulopenem etzadroxil (Orlynvah)	Beta lactam	Cell wall synthesis inhibitor	Iterum Therapeutics	IV, oral prodrug	Oral only	FDA	uUTI^##^

IV, intravenous; FDA, Food and Drug Administration; EMA, European Medicines Agency; CABP, community acquired bacterial pneumonia; ABSSSI, acute bacterial skin and skin structure infections; MHRA, Medicines and Healthcare products Regulatory Agency; CF, cystic fibrosis. ^#^Approved indications includes gram-positive and gram-negative pathogens, ^##^Aproved indications include gram-negative pathogens only.

Antimicrobial susceptibility tests (AST) including the broth microdilution assay are well characterized and are generally used to establish *in vitro* drug efficacy. The lowest concentration of an antibiotic at which bacterial growth is completely inhibited is termed the minimum inhibitory concentration (MIC). Using bacterial strain panels, the MIC_50_ (the MIC value where ≥ 50% of the strain panel is inhibited) and the MIC_90_ (the MIC value where ≥ 90% of the strain panel is inhibited) can be calculated (Schwarz et al., 2010). These values are useful benchmarks of therapeutic drug activity and where available are included herein. *In vivo* evaluation data that is publicly available was also included.

The evaluation of medical countermeasures in well-characterised animal models is fundamental, as clinical trials for these diseases may not be ethically justified. Typically, efficacy is determined in mouse models should the disease model be appropriate, and if warranted, be transitioned into higher order animal species. Parameters included in this review include survival (often the primary indicator of efficacy) and bacterial clearance in tissues (if determined). Although an attempt has been made to compare *in vivo* data sets, direct comparisons are challenging due to diverse experimental parameters (e.g., different aerobiology equipment, laboratory process differences, bacterial and animal species/strains, different challenge doses used and antibiotic dosing regimens (e.g., time of initiation, dose, and regularity of dosing).

## Antibiotics

### Finafloxacin

Finafloxacin (MerLion Pharmaceuticals) is a fifth-generation fluoroquinolone under development for the treatment of complicated urinary tract infections (cUTIs) and pyelonephritis (**Table 1**). There are three formulations available/in development, including a topical suspension which is licensed by the FDA and Health Canada for acute otitis externa. Additionally, intravenous (IV) and oral formulations have been evaluated in phase 1 and 2 clinical trials for cUTI. Finafloxacin binds to the bacterial DNA gyrase and topoisomerase IV preventing DNA replication. It is mainly differentiated from previous generations of the fluoroquinolones by its ability to retain antibacterial activity in acidic conditions, which is typical of infected body sites or in patients with acute sepsis (Higgins et al., 2010; Lemaire et al., 2011; Stubbings et al., 2011). Finafloxacin was shown to be superior to the second-generation fluoroquinolone ciprofloxacin in two cUTI/pyelonephritis clinical trials and retained potency against clinical strains shown to be resistant to ciprofloxacin (Vente et al., 2018; Wagenlehner et al., 2018).

Broad spectrum *in vitro* activity has been demonstrated for finafloxacin against *Y. pestis, F. tularensis, B. pseudomallei*, *B. mallei, B. anthracis* and *C. burnetii* at both neutral and acidic pH (Barnes et al., 2019; Peyrusson et al., 2021) (**Table 2**). The MIC_90_ values obtained for finafloxacin were low and comparable with standard-of-care antibiotics typically used as positive controls in these assays (fluoroquinolones and ceftazidime) with improved potency at acidic pH (Barnes et al., 2019). At pH 5 these were: ≤0.03 μg/mL (*Y. pestis*), 4 μg/mL (*B. pseudomallei)*, 0.5 μg/mL (*B. mallei)* and ≤0.03 μg/mL *(B. anthracis)* and at pH 7: 0.06 μg/mL (*Y. pestis*), ≤0.03 μg/mL *(F. tularensis)*, 4 μg/mL *(B. pseudomallei)*, 0.5 μg/mL (*B. mallei*) and 0.12 μg/mL (*B. anthracis)*. In addition, bactericidal activity was demonstrated in time kill assays against all of the bacterial agents, except for *C. burnetii*, where a cell culture model was used to demonstrate a 300-fold reduction in the intracellular bacterial load following finafloxacin treatment (Peyrusson et al., 2021) (**Table 2**). It has been suggested that this improved activity is due to the rapid influx of finafloxacin into cells, the accumulation of high levels within the cell and a slow efflux rate out (Chalhoub et al., 2020).

**Table 2:** A summary of the published *in vitro* and *in vivo* data for the biodefence pathogens and the antibiotics discussed in this review.

Antibiotic	Bacteria	*in vitro*				*in vivo*					Reference
		Number of strains	MIC_50_ (µg/mL)	MIC_90_ (µg/mL)	MIC_90_ range (µg/mL)	Animal model and bacterial strain	Challenge route and dose	Treatment initiation time (hpc)	Treatment dosing regimen	Survival and clearance	
Finafloxacin	*Y. pestis*	10	≤0.03 (pH5) ≤0.03 (pH7)	≤0.03 (pH5) 0.06 (pH7)	≤0.03 (pH5) ≤0.03-0.12 (pH7)	Mouse, BALB/c CO92	Nose only aerosol Mean retained dose 14 x LD_50_	24 + 38	23.1 mg/kg orally (q8h) for 3 or 7 days	100% protection for 24h (3 and 7 days). 100% and 90% for 38h (3 and 7 days respectively) at days 35–37 pc. No bacteria detected in survivors	(Barnes et al., 2019; Barnes et al., 2021)
	*F. tularensis*	10	ND (pH5) ≤0.03 (pH7)	ND (pH5) ≤0.03 (pH7)	ND (pH5) ≤0.03 (pH7)	Mouse, BALB/c Schu S4	Nose only aerosol Mean retained dose 54 x LD_50_	24 + 72	23.1 mg/kg orally (q8h) for 3 or 7 days	100% protection at 24 h (3 and 7 days). 0% and 50% (for 3 and 7 days respectively) at 72h at days 34–35 pc. No bacteria detected in survivors	(Barnes et al., 2019; Barnes et al., 2021)
	*B. pseudomallei*	21	2	4	0.12-5 (pH5) 0.5-8 (pH7)	Mouse, BALB/c K96243	Nose only aerosol Mean retained dose 21 x LD_50_	24 + 36	23.1 mg/kg orally (q8h) for 14 days	90% protection (both 24 + 36h) and bacteria in tissues of survivors at day 42–43 pc.	(Barnes et al., 2019; Barnes et al., 2022)
	*B. mallei*	10	0.12 (pH5) 0.5 (pH7)	0.5 (pH5) 0.5 (pH7)	≤0.03-0.5 (pH5) ≤0.03-0.5 (pH7)	Mouse, BALB/c 23344	Nose only aerosol 44 x LD_50_	24	37.5 mg/kg orally (q8h) for 7 days	55% protection at day 65 pc. Bacteria detected in spleens of survivors.	(Barnes et al., 2019; Barnes et al., 2022)
	*B. anthracis*	10	≤0.03 (pH5) 0.06 (pH7)	≤0.03 (pH5) 0.12 (pH7)	≤0.03-0.06 (pH5) 0.06-0.12 (pH7)	–	–	–	–	–	(Barnes et al., 2019)
	*C. burnetii*	1 (Nine Mile Phase 1)	0.03	N/A	N/A	Mouse, AJ Nine Mile (Phase 1)	Head only aerosol Inhaled dose 1.5 x 10^6^	24	30 mg/kg orally (q24h) for 7 or 14 days	No loss in body weight or development of clinical signs (for 7 and 14 days). Reduced splenomegaly and increased lung weight in survivors.	(Barnes et al., 2019; Hartley et al., 2021)
Delafloxacin	*Y. pestis*	28	0.016	0.016	0.008-0.016	–	–	–	–	–	(Frean et al., 2003)
	*F. tularensis*	–	–	–	–	–	–	–	–	–	–
	*B. pseudomallei*	30	0.5	1	0.12-2	Mouse, BALB/c 1026b	Whole body aerosol Mean inhaled dose 135 x LD_50_	16 + 24	30, 50, 80 mg/kg (q6h) SC for 21 days.	90-100% protection for 50 and 80 mg/kg and 70% for 30 mg/kg at day 62 pc. Spleens from survivors clear, no other tissues collected.	(McCurdy et al., 2021)
	*B. mallei*	–	–	–	–	–	–	–	–	–	–
	*B. anthracis*	30	≤0.001 (pH5.5) 0.002 (pH7.2)	0.001 (pH5.5) 0.004 (pH7.2)	≤0.001 (pH5.5) 0.004 (pH7.2)	Mouse, BALB/c	Whole body aerosol 103 x LD_50_	24 + 48	30, 50, 62.5 mg/kg SC	90% survival at day 30 pc with 62.5 mg/kg dose with treatment starting 24 h pc	(McCurdy et al., 2023)
	*C. burnetii*	–	–	–	–	–	–	–	–	–	
Levofloxacin	*Y. pestis*	100	<0.03	<0.03	0.03-0.06	Mouse, BALB/c CO92 NHP, AGM CO92 NHP, AGM CO92	Whole body aerosol 20 x LD_50_ Head only aerosol 3–145 x LD_50_ Head only aerosol 92 x LD_50_	24 6h following a temperature of ≥39°C for 1h 0, 18, 16, 24 post-fever (a temperature of 1.5°C above normal)	15 mg/kg IP (q12h) for 5 days daily IV infusion at 8 mg/kg followed by 2 mg/kg at 12 ± 0.5h later for 10 days 8 mg/kg followed by 2 mg/kg via a catheter for 10 days	100% protection at day 22 pc. No information on clearance. 100% protection at 28 days pc and clearance in all harvested tissues of survivors 100 and 57% protection at day 28 pc for those treated 0–20 and 20.1–30 hours after onset of fever respectively.	(Frean et al., 1996; Heine et al., 2007) (Layton et al., 2011; Campbell et al., 2020)
	*F. tularensis*	92 (Type A strains)	–	0.06	0.15-0.12	NHP, Marmoset Schu S4 Mouse, BALB/c Schu S4	Head only aerosol 300 CFU Intranasal 100 CFU	24 48, 72, 96 + 120	16.5 mg/kg orally (q12h) for 10 days 40 mg/kg IP (q24h)	100% protection at day 24 pc and clearance in all tissues of survivors. 100% protection for 48 and 72h, 80% protection for 96h. No protection at 120h. No clearance data included.	(Klimpel et al., 2008; Urich and Petersen, 2008; Nelson et al., 2010)
	*B. pseudomallei*	50	2	2	1-32	Mouse, BALB/c K96243	Nose only aerosol Mean retained dose 10 x LD_50_	6	50 mg/kg, orally (q24h) for 7 days (suboptimal)	55% protection at day 36 pc. No information on clearance	(Thibault et al., 2004; D'Elia et al., 2019)
	*B. mallei*	15	1	1	0.125-4	Mouse, BALB/c 23344	Intranasal 4.7 × 10^5^ CFU	24	20 mg/kg IP (q24h) for 7 days	100% protection at day 34 pc. Bacteria detected in spleens	(Judy et al., 2004; Thibault et al., 2004)
	*B. anthracis*	30	0.125	0.25	0.03-1	NHP, Rheus, Ames Mouse, BALB/c Ames	Head only aerosol 17–118 × LD_50_ Whole body aerosol 30.5 x LD_50_	24 48	15 mg/kg orally followed by 4 mg/kg 12h later, for 10 days. 0.75, 2.5, 5, 7.5, 10, 15, 20 mg/kg IP (q12h) for 14 days 15 mg/kg IP (q12h) for 14 days	90% protection at day 100 pc and clearance in tissues of survivors. 100% protection at day 38 pc for 5, 10, 20 mg/kg. 40, 80, 100, 100% protection for 0.75. 2.5, 7.5, 15 mg/kg respectively 60% protection at day 40 pc. Spleens were clear.	(Cavallo et al., 2002; Kao et al., 2006)
	*C. burnetii*	1 (Nine Mile Phase 1) 1 (Nine Mile Phase 2)	1 0.5-4	N/A N/A	N/A N/A	Mouse, AJ Nine Mile (Phase 1)	Head only aerosol Mean presented dose 1 × 10^7^ GE	24	40 mg/kg IP (q12h) for 7 days	Reduction of weight loss and no development of clinical signs.	(Clay et al., 2021)
Omadacycline	*Y. pestis*	30	1	1	0.12-2	Mouse, BALB/c CO92	Whole body aerosol Mean of 29.4 x LD_50_	24	5, 10, 20, 40 mg/kg ip (q12h) for 7 days	90% protection at day 41 pc for 40 mg/kg. No protection for the lower doses. Spleens clear (in 3 selected survivors).	(Steenbergen et al., 2017)
	*F. tularensis*	–	–	–	–	–	–	–	–	–	–
	*B. pseudomallei*	–	–	–	–	–	–	–	–	–	–
	*B. mallei*	–	–	–	–	–	–	–	–	–	–
	*B. anthracis*	30 53	0.03 0.015	0.06 0.03	<0.03-0.06 ≤0.008-0.25	Mouse, BALB/c Ames Mouse, BALB/c BAC’4-2	Whole body aerosol 12 and 7.6 x LD_50_ Mean of 30.5 x LD_50_ Mean of 30.5 x LD_50_ Whole body aerosol	24 48 24	5, 10, 20 mg/kg IP (q12h) for 14 days 0.75, 2.5, 7.5,15 mg/kg IP (q12h) for 14 days 15 mg/kg IP (q12h) for 14 days 0.75, 2.5, 3.75, 5, 7.5 and 15 mg/kg IP (q12h) for 14 days	100% protection at day 38 pc for all doses. Clearance from spleens, lungs colonised. 100% protection at day 40 pc for 7.5 and 15 mg/kg, 80% for 2.5 mg/kg and 40% for 0.75 mg/kg. 60% protection at day 40 pc. 100% survival at day 28 pc for 2.5, 3.75, 5 and 7.5 mgkg, 90% for 0.75 mg/kg and 80% for 15 mg/kg. Bacteria detected in the lung, spleen and blood of survivors.	(Steenbergen et al., 2017; Heine et al., 2024)
	*C. burnetii*	–	–	–	–	–	–	–	–	–	–
Gepotidacin	*Y. pestis*	138	0.25-0.5	0.5-1	≤0.008-2	NHP, AGM CO92	Head only Aerosol 25–309 x LD_50_	1–3 h post an increase in temperature of ≥1.5°C for 2h	2 daily infusions or a loading dose (ranging from 10–18 mg/kg) followed by a 2 mg/kg maintenance dose (4–6 daily infusions) via a catheter for 10 days.	100, 92, 75 and 80% protection at days 28–32 pc for 16 mg/kg (q8h), 18 mg/kg (q12h), 14 mg/kg (q12h) and 12 mg/kg (q8h). No bacteria detected in survivors.	(Jakielaszek et al., 2022)
	*F. tularensis*	Gepotidacin	0.25	0.5	0.06-4	Rats, Fischer 344 Schu S4 NHP, Cynomolgus macaque Schu S4	Aerosol Dose unknown Head only aerosol 1328 CFU	Unknown 24h ± 2h of an temperature of ≥1.5°C for 2h	Concentration unknown 14 days IV infusion of 22 mg/kg (2h loading dose) followed at 3.5h by a 4h infusion of 2 mg/kg (q8h) for a total dose of 72 mg/kg/day for 10 days	91% protection at day 28 pc. No clearance data included. 100% protection at day 43 pc and clearance in tissues from survivors.	(Jakielaszek et al., 2023)
	*B. pseudomallei*	–	–	–	–	–	–	–	–	–	–
	*B. mallei*	–	–	–	–	–	–	–	–	–	–
	*B. anthracis*	160	0.5-1	0.5-1	0.12-2	Rabbit, New Zealand Whites	Aerosol 191 x LD_50_	3-4.3 h following a positive ECL result	2h infusion (30 mg/kg) followed 1hr later by a 4h infusion of 8 mg/kg. Repeated TID every 24h for 5 days.	90.1% protection at day 28 pc. One survivor colonised in the heart, brain, kidney and mediastinal lymph node, two colonised in the lung and spleen.	(Hilliard et al., 2024)
	*C. burnetii*	–	–	–	–	–	–	–	–	–	–
Tebipenem	*Y. pestis*	29	0.03	0.03	0.0005-0.03	Mouse, BALB/c CO92	Nose only aerosol Mean inhaled dose 240 x LD_50_	12, 24 + 36	33.3 mg/kg orally (q8h) for 14 days	100, 83 and 75% protection at day 29 pc for 12, 24 and 36h. No bacteria detected in spleen, livers and lungs.	(Clayton et al., 2021)
	*F. tularensis*	29	16	>64	0.5->64	–	–	–	–	–	(Clayton et al., 2021)
	*B. pseudomallei*	29 102	2 2	2 2	1-4 NS	–	–	–	–	–	(Seenama et al., 2013; Clayton et al., 2021)
	*B. mallei*	30	0.5	1	0.25-1	–	–	–	–	–	(Clayton et al., 2021)
	*B. anthracis*	30	0.004	0.008	0.001-0.008	Mouse, BALB/c Ames	Whole body aerosol 15 x LD_50_	24	12.5, 25, 50 mg/kg orally (q8h) for 14 days	100%, 80% and 80% protection at day 34 pc for 12.5, 25, 50 mg/kg respectively. All spleens harvested (3 per group) clear, all lungs harvested (3 per group) colonised.	(Clayton et al., 2021)
	*C. burnetii*	–	–	–	–	–	–	–	–	–	–
Sulopenem	*Y. pestis*	30	0.063	0.12	0.015-0.125	–	–	–	–	–	(Dunne et al., 2021)
	*F. tularensis*	30	8	32	2-32	–	–	–	–	–	(Dunne et al., 2021)
	*B. pseudomallei*	30	1	1	1	–	–	–	–	–	(Dunne et al., 2021)
	*B. mallei*	30	0.25	0.5	0.06-0.5	–	–	–	–	–	(Dunne et al., 2021)
	*B. anthracis*	30	0.015	0.03	<0.004-0.25	Mouse, BALB/c Ames	Whole body aerosol 15 x LD_50_	24	12.5, 25, 50 mg/kg orally (q8h) for 14 days	100%, 80% and 80% protection at day 34 pc for 12.5, 25, 50 mg/kg respectively. All spleens harvested (3 per group) clear, all lungs harvested (3 per group) colonised.	(Dunne et al., 2021; Puttagunta et al., 2022)
	*C. burnetii*	–	–	–	–	–	–	–	–	–	–

MIC, minimum inhibitory concentration; ND, not determined; N/A, not applicable; NS, not stated; LD_50_, median lethal dose; AGM, African green monkeys; hpc, hours post-challenge; h, hours; pc, post-challenge; SC, subcutaneous; IP, intraperitoneal; IV, intravenous - - no data publicly available; ECL, electrochemiluminescence; TID, three times a day.

*In vivo* efficacy of finafloxacin has also been demonstrated using an orally delivered human equivalent dose in murine models of inhalational tularaemia, plague, Q fever, melioidosis and glanders (**Table 2**). Finafloxacin offered protection that was not statistically different to that afforded by ciprofloxacin and bacterial clearance when administered as treatment for plague. It was also comparable to co-trimoxazole as a treatment for glanders (**Table 2**) (Barnes et al., 2021; Barnes et al., 2022). Finafloxacin offered a significant improvement in survival compared to ciprofloxacin and doxycycline for the treatment of melioidosis and ciprofloxacin for the treatment of tularaemia (Barnes et al., 2021; Barnes et al., 2022). In a non-lethal mouse model of Q fever, finafloxacin reduced the clinical signs of infection and weight loss when compared to ciprofloxacin and doxycycline (Hartley et al., 2021).

### Delafloxacin

Delafloxacin (Melinta Therapeutics) is a fourth-generation fluoroquinolone, approved by the FDA and the EMA for the treatment of community acquired bacterial pneumonia (CABP) and acute bacterial skin and skin structure infections (ABSSSIs) (**Table 1**) (Melinta Therapeutics, 2017). Both IV and oral formulations are available, allowing for administration in both the inpatient and outpatient settings (McCurdy et al., 2023). Like finafloxacin, delafloxacin inhibits bacterial DNA gyrase and topoisomerase IV, and also has enhanced MICs at low pH, demonstrating a bactericidal effect against gram-negative and gram-positive organisms (Kocsis et al., 2021).

The MIC_90_ values obtained for *Y. pestis* (0.016 μg/mL [pH 7.2]) and *B. anthracis* (≤0.001 μg/mL [pH 5.5] and 0.04 μg/mL [pH 7.2]), are low and comparable to those obtained for the fluoroquinolone class (0.016-0.06 μg/mL) (**Table 2**) (Frean et al., 2003; McCurdy et al., 2023). The MIC_90_ for *B. pseudomallei* (1 μg/mL) is comparable to standard-of-care antibiotics. Delafloxacin also demonstrates activity against *B. pseudomallei* overexpressing RND efflux pumps such as BpeEF-OprC (McCurdy et al., 2021; McCurdy et al., 2022).

Delafloxacin has been shown to be efficacious in murine models of inhalational melioidosis and anthrax (**Table 2**). Delafloxacin afforded protection not significantly different to ciprofloxacin in mice infected with *B. anthracis* (McCurdy et al., 2023). Bacterial clearance was observed in the spleens of survivors from the anthrax study; however, lungs were colonized, likely due to spore persistence. When evaluated against inhalational melioidosis, delafloxacin offered a significant improvement in survival compared to ceftazidime and cleared colonizing bacteria from spleens (McCurdy et al., 2021).

### Levofloxacin

Levofloxacin is a third-generation fluoroquinolone licensed by the MHRA and FDA for indications including pneumonia, rhinosinusitis, chronic bronchitis, pyelonephritis, urinary tract infections and skin or skin structure infections (**Table 1**) (Podder et al., 2025). In addition, it is the only antibiotic discussed in this review which is licensed by the FDA for the treatment of *Y. pestis* and *B. anthracis* infections. Levofloxacin has the same mechanism of action as finafloxacin and delafloxacin and has broad spectrum activity against gram-negative and gram-positive organisms including methicillin resistant *Staphylococcus aureus* (MRSA), *Streptococcus pneumoniae*, *Haemophilus influenzae* and *Moraxella catarrhalis* (Croom and Goa, 2003).

The MIC_90_ values obtained for *Y. pestis*, *F. tularensis*, and *B. anthracis* are low (< 0.03, 0.06, and 0.25 μg/mL respectively) and comparable to those for other fluoroquinolones (**Table 2**) (Frean et al., 1996; Cavallo et al., 2002; Urich and Petersen, 2008). Similarly, the MIC_90_s for *B. pseudomallei* and *B. mallei* (2 μg/mL and 1 μg/mL respectively) are comparable with comparator antibiotics (Thibault et al., 2004). There is limited *in vitro* data generated for *C. burnetii*, however an MIC of 1 μg/mL has been reported for strain Nine Mile (Phase I) with an intracellular MIC of 0.16 μg/mL (Clay et al., 2021; Hartley et al., 2021).

*In vivo* efficacy studies delivering the antibiotic by the IV and oral routes have been performed in murine and non-human primate (NHP) models (**Table 2**). Levofloxacin completely protected animals and cleared bacteria from tissues in an African Green Monkey (AGM) model of plague and a marmoset model of tularaemia (Nelson et al., 2010; Layton et al., 2011). Delaying treatment resulted in a reduction in survival in the AGM (Campbell et al., 2020). High levels of protection and clearance was also demonstrated in a rhesus macaque model of anthrax treated with levofloxacin (Kao et al., 2006).

Levofloxacin provided complete protection when delivered early in a murine model of plague (Heine et al., 2007). High levels of protection were demonstrated when levofloxacin was delivered following an intranasal challenge of *F. tularensis* and *B. mallei* (Judy et al., 2004; Klimpel et al., 2008). Limited information is available for the *in vivo* evaluation of *B. pseudomallei* infections with levofloxacin as fluoroquinolones are not clinically recommended for melioidosis; however, 55% survival was reported when a suboptimal course of levofloxacin was initiated at 6 hours post-challenge in a mouse model (D'Elia et al., 2019). Levofloxacin delivered by the intraperitoneal route reduced weight loss and the development of clinical signs of disease in a mouse model of Q fever (Clay et al., 2021).

### Omadacycline

Omadacycline (Paratek Pharmaceuticals) is a first-in-class aminomethylcycline of the tetracycline family, approved by the FDA in 2018 for the treatment of CABP and ABSSSIs (Watkins and Deresinski, 2019) (**Table 1**). In addition, it is the first once-daily multi-indication oral antibiotic to be approved by the FDA in 10 years (Watkins and Deresinski, 2019). Both oral and IV formulations are available. Mechanistically, it binds the 30S ribosomal subunit, preventing the binding of aminoacyl-tRNA and inhibiting protein synthesis. Omadacycline is active against a wide range of pathogens including MRSA, vancomycin resistant *Enterococcus* and penicillin resistant *S. pneumoniae* (Tanaka et al., 2016).

The MIC_90_ obtained for *Y. pestis* (1 μg/mL), which, whilst higher than the previously discussed fluoroquinolones, is within the range of susceptible gram-negative pathogens for the class (**Table 2**) (Steenbergen et al., 2017). The MIC_90_ for *B. anthracis* has been reported as 0.06 μg/mL and 0.03 μg/mL which is comparable to previous generations of the fluoroquinolones (Steenbergen et al., 2017; Heine et al., 2024). Omadacycline also demonstrated high potency against the ciprofloxacin-resistant strain of Ames (BAC’4-2) (Heine et al., 2024).

*In vivo* efficacy delivering the antibiotic by the IP route has also been demonstrated in murine models of inhalational plague and anthrax (**Table 2**). Omadacycline was shown to offer an equivalent level of protection to ciprofloxacin when administered as treatment for infection with *Y. pestis* (Steenbergen et al., 2017). Bacterial clearance was observed in spleens. When evaluated against infection with *B. anthracis*, omadacycline also offered an equivalent level of protection to ciprofloxacin (**Table 2**) (Steenbergen et al., 2017). Spleens were clear from colonizing bacteria in survivors. In a separate study, omadacycline provided complete protection in an inhalational anthrax mouse model with strain BAC’4-2 (Heine et al., 2024).

### Gepotidacin

Gepotidacin (GSK) is a bactericidal first-in-class triazaacenaphthylene that was recently approved by the FDA for the treatment of uncomplicated UTIs (uUTIs) (Wagenlehner et al., 2024) (**Table 1**). It is also in development for the treatment of gonorrhoea and both oral and IV formulations have been produced. Gepotidacin inhibits bacterial DNA gyrase and the type IIA topoisomerase at a site and mechanism distinct from the fluoroquinolones. As the first approved novel bacterial topoisomerase inhibitor (NBTI), gepotidacin is of interest as its potency is not impaired by the on-target mutations associated with fluoroquinolone resistance. Two phase 3 clinical trials evaluating gepotidacin as a therapeutic for uUTIs were stopped early due to the superiority of results obtained, leading to the FDA approving the use for the treatment of uUTIs in female adults and paediatric patients over 12 (GSK, 2022; GSK, 2025). Gepotidacin has demonstrated *in vitro* activity against gram-positive and gram-negative organisms, including MRSA, *Shigella* species, *S. pneumoniae* and *Mycobacteria* (Biedenbach et al., 2016; Ahmad et al., 2022).

Potency has been demonstrated *in vitro* for gepotidacin against *Y. pestis*, *F. tularensis*, and *B. anthracis*, all with MIC_90_ values between 0.5 and 1 μg/mL (**Table 2**) (Jakielaszek et al., 2022; Jakielaszek et al., 2023; Hilliard et al., 2024). It is worth noting that the *in vitro* MIC screening with gepotidacin utilised large panels of bacterial strains (120+), which is impressive. It also retained activity against aminoglycoside and doxycycline resistant mutants of *Y. pestis* and fluoroquinolone resistant mutants of *B. anthracis* (Jakielaszek et al., 2022; Hilliard et al., 2024).

Several studies utilizing large animal models have been published that demonstrate the efficacy of gepotidacin against *Y. pestis*, *F. tularensis*, and *B. anthracis*. This includes *in vivo* efficacy data in NHP models of plague and tularaemia where fever was used as a trigger-to-treat (**Table 2**). Gepotidacin provided a high level of protection (75-100%) and bacterial clearance in an AGM model of inhalational plague, irrespective of the antibiotic dose and dosing regimen (Jakielaszek et al., 2022). There were no differences between the level of protection offered in relation to the number of doses of antibiotic administered. This is similar to the data previously generated for ciprofloxacin and levofloxacin in this NHP model (Layton et al., 2011; Campbell et al., 2020). When administered to cynomolgus macaques following an inhalational *F. tularensis* exposure, gepotidacin provided complete protection and bacterial clearance (Jakielaszek et al., 2023). This is similar to data generated with levofloxacin in a marmoset model of tularaemia (Nelson et al., 2010). Gepotidacin was also shown to be 90% protective in a lethal, trigger-to-treat New Zealand white rabbit model of inhalational anthrax (Hilliard et al., 2024).

### Tebipenem

Tebipenem pivoxil hydrobromide (Spero Therapeutics) is an oral carbapenem prodrug being developed for the treatment of cUTIs (**Table 1**). Carbapenems are bactericidal agents that enter the periplasm space and acylate penicillin-binding proteins (PBPs). This weakens the peptidoglycan of the cell wall which lyses the bacterial cell (Mahalingam and Shenoy, 2020). Traditionally, carbapenems have only been available for IV administration; therefore, the potential to leverage carbapenem activity in an orally-available drug would be significant. Tebipenem has been evaluated in a phase 3 clinical trial for the treatment of cUTIs and pyelonephiritis; however, the FDA has requested further data to be generated and submitted before considering licensure. Tebipenem is active against gram-negative and gram-positive organisms including extended spectrum β-lactamase (ESBL) and AmpC β-lactamase producing *Klebsiella pneumoniae, Escherichia coli*, *Proteus* spp, and MRSA (Cotroneo et al., 2020).

The MIC_90_ values obtained for *Y. pestis*, *B. pseudomallei, B. mallei* and *B. anthracis* are low (0.03, 2, 1 and 0.008 μg/mL, respectively (**Table 2**)) (Seenama et al., 2013; Clayton et al., 2021). The MIC for a ciprofloxacin resistant Ames strain of *B. anthracis* was similar (0.008 μg/mL). There was no measurable *in vitro* activity for *F. tularensis* (MIC_90_ of > 64 μg/mL), which is consistent with the activity of other carbapenems (Caspar and Maurin, 2017).

Oral tebipenem has been evaluated in murine models of pneumonic plague and inhalational anthrax (**Table 2**). It offered an equivalent level of protection to ciprofloxacin when administered as treatment for infection with *Y. pestis* (Clayton et al., 2021). Bacterial clearance was observed in lungs, livers and spleens. When evaluated against an infection with *B. anthracis*, tebipenem also offered an equivalent level of protection to ciprofloxacin (Clayton et al., 2021). Spleens were clear at the end of the study with lungs colonized.

### Sulopenem

Sulopenem (Iterum Therapeutics) is a broad spectrum thiopenem β-lactam, being developed for the treatment of infections caused by multi-drug resistant bacteria (**Table 1**). Two formulations are currently being evaluated, an orally-available prodrug (sulopenem etzadroxil) or sulopenem for IV administration. Sulopenem retains many characteristics of the carbapenem family and shares the same mechanism of action (Zhanel et al., 2022). It has been evaluated in multiple phase clinical 3 trials for the treatment of uUTIs, cUTIs and pyelonephritis and is active against gram-negative and gram-positive organisms including penicillin resistant *S. pneumoniae* and *H. influenzae* and *M. catarrhalis* strains able to produce β-lactamases (Butler et al., 2023; Dunne et al., 2023). It was recently approved by the FDA to treat uUTIs in adult women with limited or no alternative oral antibacterial treatment options (delivered with the renal tubular transport inhibitor probenecid) (FDA, 2024).

The MIC_90_ values obtained for *Y. pestis*, *B. pseudomallei, B. mallei* and *B. anthracis* are low (0.12, 1, 0.5 and 0.03 μg/mL, respectively and similar to carbapenems (Dunne et al., 2021) (**Table 2**). Like tebipenem, there is limited *in vitro* activity for sulopenem against strains of *F. tularensis* (MIC_90_ of 32 μg/mL). Sulopenem has been evaluated for efficacy in a murine model of inhalational anthrax where it offered an equivalent level of protection to ciprofloxacin (**Table 2**) (Puttagunta et al., 2022). Spleens were clear of bacteria at the end of the study with lungs colonized.

## Conclusions

The identification and evaluation of novel broad spectrum medical countermeasures antibiotics for the treatment of the diseases caused by the bacterial pathogens of biodefence interest remains a significant priority to both military and public health. This review discusses several antibiotics that are in advanced clinical development that, although not being developed for this purpose, have demonstrated efficacy against these pathogens, and offer potential alternatives or improvements to first-line therapies. Novel or newer generations of antibiotics such as those discussed here bring innovative tools to fight an increasingly variable biothreat landscape. Robust preclinical evaluation of candidates provides *in vitro* and *in vivo* efficacy data that can support regulatory approval or be leveraged in an emergency to rapidly identify alternative therapies. Continued work is needed to ensure the most appropriate and effective therapies are prepositioned to combat these virulent pathogens.

## Footnotes

1 Priority pathogen families research and development tool

2 Federal Select Agent Program
